# Don’t fear the reaper: Tracing the organized breakdown of dying cells in the lateral root cap

**DOI:** 10.1093/plcell/koad321

**Published:** 2023-12-23

**Authors:** Sonhita Chakraborty

**Affiliations:** Assistant Features Editor, The Plant Cell, American Society of Plant Biologists; Department of Forest Genetics and Plant Physiology, Swedish University of Agricultural Sciences, Umeå Plant Science Centre, Linnaeus väg 6, 907 36 Umeå, Sweden

Is there life after death? The answer is a resounding yes for the developing Arabidopsis root cap. Although death might seem like the polar opposite of growth, the timely demise and subsequent removal of older cells at the lateral root cap (LRC) is an essential part of root growth and differentiation. When it is time to die, the terminally differentiated cell prepares itself by transcriptionally activating relevant genes. The transcription factor *SOMBRERO* controls programmed cell death (PCD) as a part of the LRC differentiation program to maintain proper root cap size (Fendrych et al. 2014). After preparation at the genetic level, a “death sentence” trigger initiates a cascade of events for the execution of PCD. Internal structures like the vacuole collapse, caustic enzymes are released, the cytosol acidifies, and the plasma membrane (PM) becomes more permeable. Finally, the leftover cellular debris is cleared to make room for differentiating cells (Huysmans et al. 2017). When older LRC cells that have served their purpose do not undergo PCD, they hinder developing cells from differentiating and alter LRC size (Fendrych et al. 2014).

We have only scratched the surface of this highly intricate process, and many outstanding questions about developmental PCD (dPCD) at the LRC remain unanswered: What is the triggering stimulus that initiates the cascade of dPCD events at the LRC in the first place? Is there a specific order in which the cellular components decompartmentalize? Which of these events are under the control of *SMB*? Using a combination of live-cell imaging, transmission electronic microscopy, and pharmacological manipulation of wild-type, mutant and transgenic lines, **Jie Wang and colleagues** (Wang et al. 2024) connect the dots and map the spatio-temporal sequence of events that occur during dPCD at the LRC (Figure).

**Figure.**
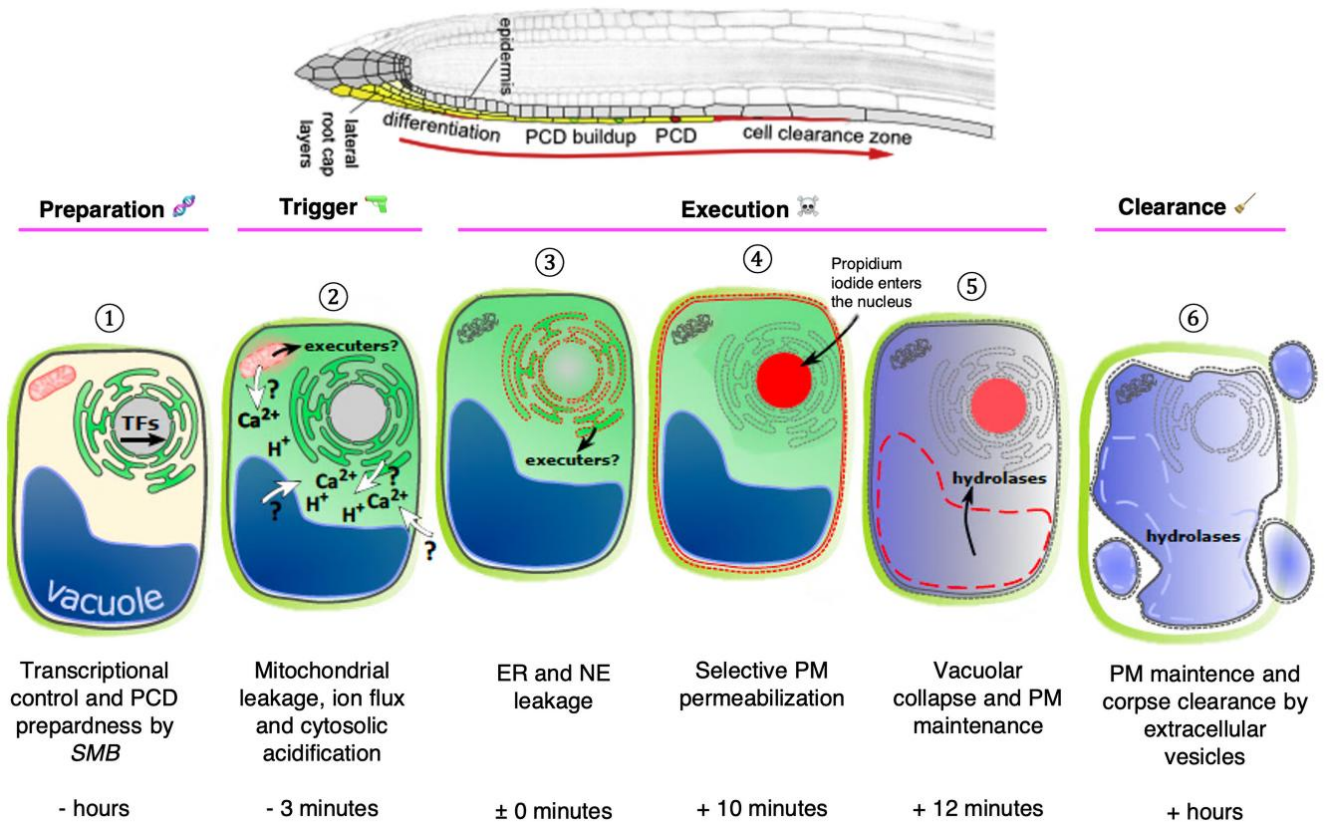
An overview of the events that occur during PCD at the LRC. (1) PCD preparation starts with transcriptional control by *SMB.* (2) Ion flux from unknown sources serves as a trigger for the execution of PCD and is accompanied by mitochondrial leakage. (3) Concurrent leakage of the endoplasmic reticulum and nuclear envelope is followed by (4) selective PM permeabilization and entry of nonmembrane permeable propidium iodide. (5) Vacuolar collapse within an intact PM gives way to (6) post-PCD corpse clearance by blebbing extracellular vesicles. Adapted and modified from Fendrych et al. (2014), Graphical Abstract & Wang et al. (2024), Figure 8.

Rapid calcium (Ca^2+^) influx into the cytosol and its acidification is a prerequisite to dPCD (Wilkins et al. 2015). Using ratiometric biosensors, the authors find that transient increases in cytosolic protons (H^+^) and Ca^2+^ levels are necessary and sufficient to trigger death in LRC cells. By subsequently tracing the diffusion of markers out of their cellular compartments, the authors find that the first organelle to disintegrate is the mitochondria, followed by the near simultaneous collapse of the endoplasmic reticulum and nuclear envelope. Rupture of the hydrolase-rich vacuole occurs several minutes later followed by the clearance of post-PCD debris by extracellular vesicles (Wang et al. 2024). Each sequential decompartmentalization process is impaired or delayed in the *smb-3* mutant, which underscores the importance of *SMB* as an upstream regulator of dPCD and LRC differentiation.

Fendrych et al. (2014) previously noticed that cellular acidification is followed by increased permeability of the PM. Now, Wang et al. (2024) note that although the PM is modified and sheds membrane-bound proteins from its cytosolic side, its permeability is selective and partial even after late-stage vacuolar collapse. They go on to posit that the intact plant PM during PCD might serve to quarantine caustic enzymes away from healthy cells. The observation of an impermeable PM during plant PCD akin to the maintenance of animal apoptotic PM is surprising and exciting in the light of controversies on how PCD differs between the two kingdoms (Minina et al. 2021).

The Arabidopsis LRC is the site of an important dPCD-based differentiation program that can be easily manipulated, imaged, and studied. After much systematic experimentation of root cap cells, Wang et al. (2024) put forth a simple yet elegant model for dPCD at the LRC that is initiated by cytosolic acidification, progresses with gradual and ordered cellular decompartmentalization within an intact PM, and ends with postmortem clearance (Figure). These dPCD events are genetically under the control of the transcription factor *SMB*. The discovery of genetic regulators that work in concert with *SMB* will provide a more comprehensive understanding of the molecular mechanisms and timing of each decompartmentalization event. Needless to say, this work brings us closer to the “root” of dPCD signaling by disentangling the myriad of cellular events.

## Dive Curated Terms

The following phenotypic, genotypic, and functional terms are of significance to the work described in this paper:

SMB Gramene: AT1G79580
SMB Araport: AT1G79580
